# Genetic analysis of the rice jasmonate receptors reveals specialized functions for *OsCOI2*

**DOI:** 10.1371/journal.pone.0291385

**Published:** 2023-09-08

**Authors:** Hieu Trang Nguyen, Mohamad Cheaib, Marie Fournel, Maelle Rios, Pascal Gantet, Laurent Laplaze, Soazig Guyomarc’h, Michael Riemann, Thierry Heitz, Anne-Sophie Petitot, Antony Champion

**Affiliations:** 1 DIADE, IRD, Univ Montpellier, Montpellier, France; 2 IBMP, CNRS, Univ Strasbourg, Strasbourg, France; 3 Karlsruhe Institute of Technology, Botanical Institute, Karlsruhe, Germany; SativaGen, UNITED STATES

## Abstract

COI1-mediated perception of jasmonate is critical for plant development and responses to environmental stresses. Monocots such as rice have two groups of *COI* genes due to gene duplication: *OsCOI1a* and *OsCOI1b* that are functionally equivalent to the dicotyledons *COI1* and *OsCOI2* whose function remains unclear. In order to assess the function of *OsCOI2* and its functional redundancy with *COI1* genes, we developed a series of rice mutants in the 3 genes *OsCOI1a*, *OsCOI1b* and *OsCOI2* by CRISPR Cas9-mediated editing and characterized their phenotype and responses to jasmonate. Characterization of *OsCOI2* uncovered its important roles in root, leaf and flower development. In particular, we show that crown root growth inhibition by jasmonate relies on *OsCOI2* and not on *OsCOI1a* nor on *OsCOI1b*, revealing a major function for the non-canonical *OsCOI2* in jasmonate-dependent control of rice root growth. Collectively, these results point to a specialized function of *OsCOI2* in the regulation of plant development in rice and indicate that sub-functionalisation of jasmonate receptors has occurred in the monocot phylum.

## Introduction

Jasmonoyl-isoleucine (JA-Ile), the bioactive form of jasmonic acid (JA), regulates many physiological and developmental processes in plants such as flower and root development [1, 2]. Plants from monocot and eudicot lineages accumulate JA-Ile in response to stresses, such as injury or insect attack [3, 4]. Jasmonate perception and transcriptional regulation of JA-responsive genes relies on a conserved core protein complex, the main components of which are the F-box protein CORONATINE INSENSITIVE1 (COI1) protein, the JASMONATE ZIM DOMAIN (JAZ) transcriptional repressors and transcription factors (TFs) [5–9]. In the absence of JA-Ile, target gene expression is repressed due to TF and JAZ protein interaction within the TOPLESS (TPL)-NOVEL INTERACTORS OF JAZ (NINJA) repressor complex [10]. Upon JA-Ile accumulation, JAZ proteins are recruited by COI1, ubiquitinated and rapidly degraded in a proteasome-dependent manner, thus freeing TFs and leading to the de-repression of JA-responsive genes and subsequent physiological responses.

In eudicot species such as Arabidopsis and tomato, JA-Ile is perceived by a single receptor encoded by *COI1*, that interacts with members of the JAZ family [5–7]. In contrast, monocots such as rice, maize and wheat have two groups of *COI* genes due to gene duplication [4, 11, 12]. In rice, there are three *COI* genes, named *OsCOI1a*, *OsCOI1b*, and *OsCOI2*. *OsCOI1a* and *OsCOI1b* are orthologous to *AtCOI1* as shown by complementation of the loss-of-function *coi1-1* mutant in Arabidopsis [13]. Several studies in rice demonstrated the involvement of *OsCOI1a* and *OsCOI1b* in plant defence. Transgenic rice plants with a reduced expression of both *OsCOI1a* and *OsCOI1b* genes display a phenotype similar to gibberellic acid over-accumulating plants, including elongated internodes which suggests that jasmonate signaling promotes defence response over growth [14]. In a second study, silencing of *OsCOI1a* and *OsCOIb* gene expression led to an increased susceptibility to the rice leaf-folder insect [15] and to the rice stripe virus, the resistance to which is known to be conferred by JA signalling elements [16].

In contrast, very little is known about *OsCOI2* function in rice. OsCOI2 fails to rescue the fertility and defence response defect of *coi1-1* Arabidopsis plants, nor does it interact with any AtJAZ co-receptor [13], suggesting that OsCOI2 may have as-yet unidentified function(s) distinct from that of OsCOI1. Similarly, in maize (*Zea mays*), three closely related *ZmCOI1* genes could complement the *coi1-1* mutation in *Arabidopsis*, but *ZmCOI2* did not [11]. Interestingly, a substitution in OsCOI2 of one amino acid predicted to be involved in JA-Ile interaction allowed it to interact with AtJAZs and to rescue *coi1-1* deficiency [13]. Monocot and dicot lineages split about 140 to 150 million years ago [17]. Phylogenetic analysis revealed that OsCOI2 shares higher similarity to COI2 proteins from other monocots than to COI1 proteins from monocots and eudicots, suggesting that the monocot-specific COI1-COI2 duplication and divergence occurred early in the monocot phylum [11]. This high similarity in the sequence of COI2 proteins from monocot species, and the failure of *OsCOI2* and *ZmCOI2* to complement the Arabidopsis *coi1-1* mutant raised fundamental questions about the conservation of function of *COI2* and whether it was involved in jasmonate signalling or not. Here genetic analysis allowed us to address in parallel the functions of OsCOI1a/b and OsCOI2, and to uncover the unique roles of OsCOI2 in jasmonate-dependent developmental processes.

## Materials and methods

### Edited rice lines

To generate the JA perception mutants *oscoi1a/b* and *oscoi2*, the CRISPR-Cas9 system was used on the background cultivar Kitaake (*Oryza sativa* L. ssp. japonica) as described in [18]. Briefly, the CRISPR Guides tool on Benchling platform (https://benchling.com/crispr) was used to design the gRNAs. One gRNA was designed for each gene, *OsCOI1a* and *OsCOI1b*, to obtain the *oscoi1a/b* double mutants and two different pairs of gRNAs were designed to obtain the *oscoi2* mutants. The sequences of the gRNAs were first inserted into the pUC57-sgRNA vector, then transferred by LR recombination into the binary vector pOS-Cas9 [19]. The pOS-Cas9-gRNAs vectors were introduced in the *Agrobacterium tumefaciens* strain EHA105 by electroporation. Rice embryogenic calli were transformed via *Agrobacterium*-mediated transformation protocols. The regenerated plants were checked for transformation, by PCR using T-DNA specific primers (HPT and Cas9, S1 Table in S1 File), and for mutation, by PCR using primers flanking the targeted sequences (S1 Table in S1 File) and sequencing (Eurofins). Edited plants were transferred to a greenhouse for multiplication. Edited lines were counter-selected from T1 generation to obtain T3 homozygous lines without T-DNA insertion, except for *oscoi2-1* for which the limited number of seeds prevented such selection.

### Plant phenotyping

Seeds were sterilized in 70% ethanol for 1 minute, then in 40% commercial bleach for 30 minutes, and were rinsed 6 times in sterilized water. Disinfected seeds were then germinated on half-strength Murashige & Skoog (½MS) medium including Gamborg B5 vitamins (Duchefa Biochemie BV, Haarlem, the Netherlands) supplied with 0.7% plant agar (Duchefa) for 6 days. Plantlets of uniform size were transferred into soil and grown in a greenhouse under a 16h-day/8h-night photoperiod, at 28°C/24°C with a 75% relative humidity. Tiller and internode lengths were measured at the mature stage on the 3 highest tillers from 18 T3 homozygous lines and wild type (WT) plants. Fertility rate was measured on the same tillers as the ratio between the number of fertile spikelets and the total number of spikelets. Leaf necrotic lesions and adventitious roots were observed under an Axiozoom microscope (Zeiss). Spikelet and anther morphology were observed under a stereo microscope (Nikon SMZ1500).

### Hormone treatments

Seeds were sterilized and sown as described above. Two days after germination, seedlings with both coleoptile and primary root were transferred into glass tubes containing 20 ml of ½MS including Gamborg B5 vitamins supplied with 0.2% phytagel (Sigma) and grown in a growth chamber at 26°C under a 12h-day/12h-night photoperiod with a 70% relative humidity. For phenotyping, 5 μM JA (Sigma), 0.5 μM COR (Sigma) both dissolved in DMSO, or DMSO only (control plants) were added to the media just before pouring. Eight days after transplanting, length of the crown roots was measured for each plant. For each line, 20–24 plants were used except for the *oscoi2-1* line for which very few seeds were available. Percentages of root growth inhibition were determined by randomly comparing the length values of the treated plants to those of control plants.

For gene expression analyses, plants were transferred 4 days after transplantation from the tubes into liquid media containing either 5 μM JA or DMSO for control plants. Crown root tips (around 1 cm) from 20–24 plants were collected 6 hours after and immediately frozen in liquid nitrogen. Five independent biological samples were collected and analysed.

### Gene expression analyses

Total RNAs were extracted from rice root samples using the RNeasy Plant mini kit (Qiagen, France), with addition of an on-column DNase I digestion. First-strand cDNAs were synthesized from 1 μg of total RNA in 20 μl final volume using an oligo-dT(18)-MN primer (Eurogentec, France) and the Omniscript RT kit (Qiagen). Specific primers were designed from *O*. *sativa* Nipponbare sequences using Primer3Plus (https://primer3plus.com/) (S1 Table in S1 File) and checked for specificity on *O*. *sativa* Kitaake sequences. Quantitative-PCR assays were performed on cDNAs samples (diluted 1/50e) in an Mx30005P thermal cycler (Stratagene, USA) using the Brilliant III Ultra-fast SYBR^®^ Green QPCR Master mix with low ROX (Agilent, Santa Clara, CA, USA). Amplifications were performed in duplicate from the five biological samples. The *EXP* gene (LOC_Os06g11070) was used as a reference gene to normalize data. Relative gene expression levels were calculated using the 2^-ΔΔCt^ method.

### Statistical analyses

Experimental data were analysed using GraphPad Prism (version 9.3.0). Values were considered statistically significant when *p* ≤ 0.05.

## Results

### *OsCOI2* regulates plant development and its function is not redundant with that of *OsCOI1a* and *OsCOI1b*

**Fig 1:**
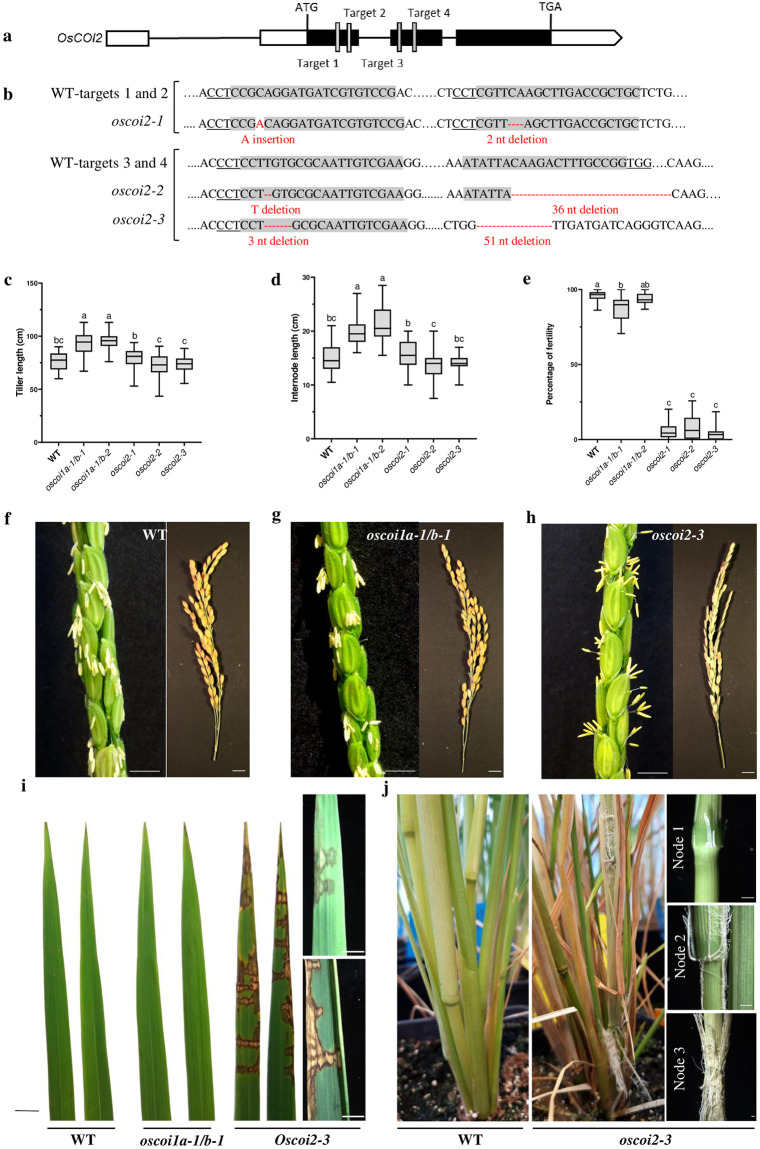
*OsCOI2* regulates vegetative and reproductive developmental programmes in rice distinct from *OsCOI1a/b*. **a**, *OsCOI2* gene structure and CRISPR-Cas9 target sites. **b**, insertion and deletion sites of three allelic mutations (*oscoi2-1*, *oscoi2-2* and *oscoi2-3*) generated by two different pairs of gRNA. **c**, quantification of tiller length and **d**, quantification of internode length. Letters indicate significant differences between lines (45<n<54, One way-ANOVA with Tuckey’s multiple comparisons test, *p*<0.05). **e**, Percentage of fertile spikelet. Letters indicate significant differences between lines (43<n<53, Kruskall-Wallis multiple comparisons test, *p*<0.05). In the boxplots, whiskers denote minimum/maximum values, the box defines the interquartile range and the centre line represents the median. (c, d, e). **f**-**h** images of WT (**f**), *oscoi1a-1/b-1*(**g**) and *oscoi2-3* (**h**) panicles. **i**, flag leaves. **j**, nodes. (**f**-**j**) Scale bar = 1 cm.

To investigate *COI2* functions in monocots, CRISPR-Cas9 technology was used to generate *oscoi2* mutant alleles in rice. Two CRISPR-Cas9 constructs targeting the first (target #1 and #2), and the second exon (target #3 and #4) of *OsCOI2*, respectively, were used to generate edited lines in the rice cultivar Kitaake (Fig 1a). Three homozygous mutant lines, named *oscoi2-1*, *oscoi2-2* and *oscoi2-3*, were isolated and further characterized. The alleles *oscoi2-1* and *oscoi2-2* include short nucleotide insertions and deletions in the first and second exon leading to a translational frame shift and premature stop codon truncating 467 amino acids and 372 amino acids in the predicted proteins, respectively. The third allele *oscoi2-3* displays fragments deleted in exon 2 eliminating 18 amino acids in the LRR domain, which can affect the protein stability (Fig 1b) [20]. In order to compare their phenotype with that of *oscoi2* mutants and assess the putative functional redundancy between OsCOI2 and OsCOI1, we also generated two double *oscoi1a oscoi1b* mutant lines (thereafter named *oscoi1a-1/b-1* and *oscoi1a-1/b-2*) by CRISPR-Cas9 (S1 Fig in S1 File).

Phenotypically, the *oscoi1a-1/b-1* and *oscoi1a-1/b-2* double mutant lines exhibited increased plant height in comparison with *oscoi2* single mutant and WT plants (Fig 1c, S2 and S3 Figs in S1 File). In addition, internode length was increased in the *oscoi1a-1/b-1* and *oscoi1a-1/b-2* compared to WT and *oscoi2* mutant lines (Fig 1d, S2 Fig in S1 File). These *oscoi1a/b* phenotypes are similar to those reported for the rice *coi1-RNAi* lines in which both *OsCOI1a* and *OsCOI1b* expression is reduced [14]. Thus, we used these new *oscoi1a-1/b-1* and *oscoi2* knockout lines to compare OsCOI1a/b and OsCOI2-mediated regulation of plant development and JA signalling in rice.

The most prominent phenotype observed in *Arabidopsis coi1-1* and tomato *jai1-1* JA receptor mutants is sterility [5, 21], but earlier studies reported only a weak reduction in fertility in *oscoi1-RNAi* and *oscoi1b*-T-DNA rice mutant lines [14, 22]. Consistent with these reports, seed-setting in our *oscoi1a/b* double mutant lines was close to WT level (Fig 1e–1g). In contrast, all three of our independent *oscoi2* mutant alleles conferred reduced seed setting (Fig 1e and 1h). None of the *oscoi1a/b*, nor *oscoi2* mutant lines displayed any abnormality in floret architecture (S4 Fig in S1 File); however, most of the *oscoi2* anthers did not dehisce (Fig 1f–1h and S4 Fig in S1 File). Hence, *OsCOI2*, rather than *OsCOI1a* or *OsCOI1b*, plays an important role during anther dehiscence stage in rice reproductive development. Importantly, even though *OsCOI2* does not restore fertility in the *Arabidopsis coi1-1* mutant background [13], our results indicated that *OsCOI2* is an essential factor for rice plant fertility, probably via jasmonate-dependent regulation of flower or seed development.

During the vegetative to reproductive transition, *oscoi2* mutant lines exhibited three distinct phenotypes not observed in WT or in *oscoi1a/b* plants. First, all three *oscoi2* mutant alleles showed spontaneous lesions on flag leaves and the last leaf mimicking disease symptoms, despite plants not being infected (Fig 1i). Second, when the main *oscoi2* panicle started to flower, above-ground adventitious roots emerged from node 1 to node 3, which resembles adventitious rooting induced by flooding in deep-water rice [23]. Third, 25 to 35% of the plants showed leaf rolling followed by tiller senescence and eventually plant death without imposing any water stress (S5 Fig in S1 File). Collectively, these data reveal that *OsCOI2* regulates plant development and that its function is not redundant with that of *OsCOI1a* and *OsCOI1b* jasmonate receptors.

### *OsCOI2* is required for JA perception in rice crown roots

**Fig 2:**
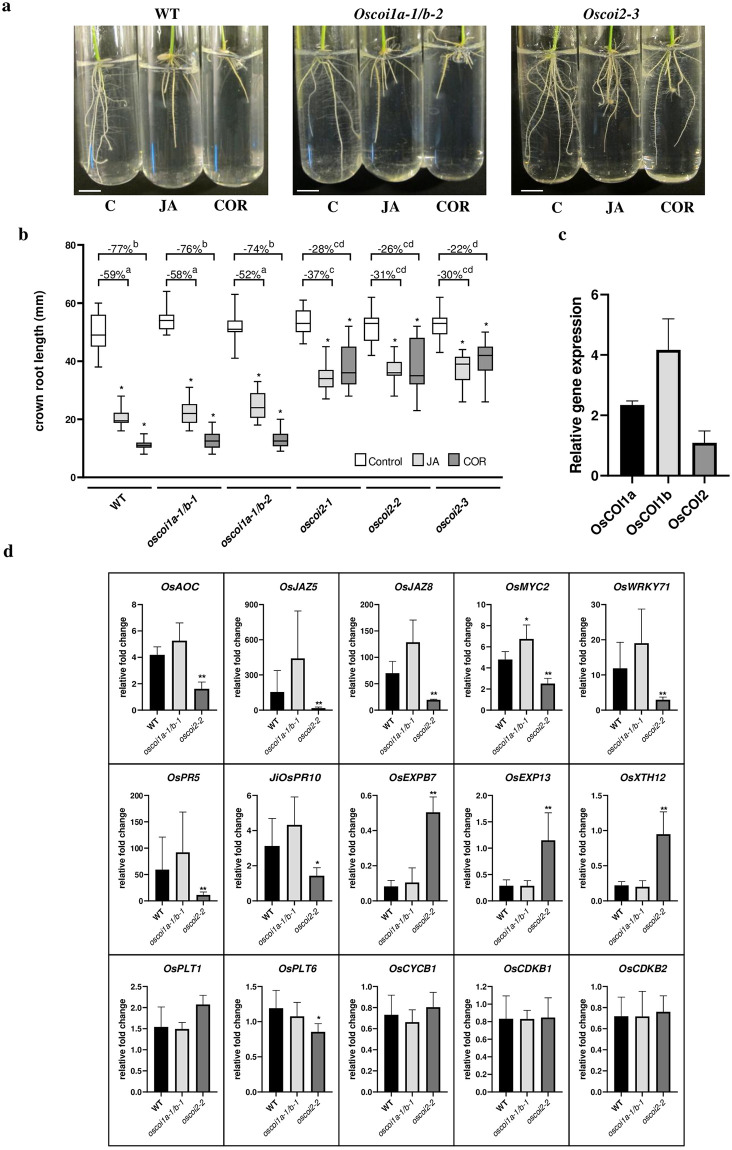
*OsCOI2* regulates jasmonate response in crown roots. **a**, Phenotype of the (WT) and two allelic mutations (*oscoi1a-1/b-2* and *oscoi2-3*) of roots in response to jasmonic acid (JA, 5 μM), coronatine (COR, 0.5 μM) and mock control (C, DMSO). Scale bars = 1 cm. **b**, effect of JA and COR compared to the mock treatment (DMSO) on crown root length in WT *oscoi1a/b* (*oscoi1a-1/b-1* and *oscoi1a-1/b-2*) and *OsCOI2* (*oscoi2-1*, *oscoi2-2* and *oscoi2-3*) mutant lines. In the boxplots, whiskers denote minimum/maximum values, the box defines the interquartile range and the centre line represents the median. Asterisks indicate significant differences between treated and control plants (20<n<24 except for *oscoi2-1* where 7<n<21, One way-ANOVA with Bonferroni’s multiple comparisons test, *p*<0.05). The percentages of crown root growth inhibition by JA and COR treatments are indicated above the respective plots from the WT, *oscoi1a-1/b-1*, *oscoi1a-1/b-2*, *oscoi2-1*, *oscoi2-2* and *oscoi2-3* mutants. Letters indicate significant differences between lines and treatments (21<n<24, One way-ANOVA with Tuckey’s multiple comparisons test, *p*<0.05). **c**. expression of *OsCOI1a*, *OsCOI1b* and *OsCOI2* genes in crown root tips from wild-type plants. Data are presented as the means +/- SD from five biological replicates. **d**, regulatory effects of JA on the transcription of jasmonate responsive genes, cell elongation and division and defence genes. Data are presented as means +/- SD from five biological replicates. Asterisks indicate significant differences between the WT and the double *oscoi1a-1/b-1* mutant or the *oscoi2-2* mutant (Mann-Whitney tests, * *p* <0.05, **p<0.01).

Next we tested the hypothesis that *OsCOI2* participates in JA signalling in rice. Inhibition of seedling root growth by jasmonate or jasmonate analogue treatments has been extensively used as a bioassay to identify jasmonate response mutants in plants [5, 21, 24]. Under our growth conditions, JA treatment at 5 μM inhibited significantly WT crown root growth (S6 Fig in S1 File). Interestingly, at this concentration, JA also inhibited root growth of *oscoi1a/b* double mutant plants to the same extent as in the WT (Fig 2a and 2b). In contrast, crown root growth was significantly less sensitive to exogenous JA treatment in *oscoi2-1*, *oscoi2-2*, and *oscoi2-3* genetic backgrounds (Fig 2a and 2b and S7 Fig in S1 File). Similarly, coronatine (COR), a bacterial mimic of JA-Ile, strongly inhibited root growth of WT and *oscoi1a/b* lines but had a significantly weaker effect on root growth of *oscoi2* mutant alleles (Fig 2a and 2b).

To explore the molecular mechanisms associated with inhibition of crown root growth by jasmonate, we focused on crown root tips, where cell proliferation and elongation occur. All three JA receptor genes *OsCOI1a*, *OsCOI1b and OsCOI2* were expressed in crown root tips (Fig 2c). Expression of the *OsCOIa* and *OsCOI1b* was similar in WT and *oscoi2* mutant crown root tips thus indicating that no genetic compensation occurred (S8 Fig in S1 File). Similarly, *OsCOI2* expression was not different in WT and *oscoi1a-1/b-1* plants.

Induction of jasmonate responsive genes related to JA biosynthesis (*OsAOC*) and signalling (*OsJAZ5*, *OsJAZ8* and *OsMYC2*) by exogenous JA application was compromised in *oscoi2-2* crown roots relative to WT, but not in the *oscoi1a-1/b-1* mutant background (Fig 2d, top panels). Consistent with the crown root growth phenotype, expression of the cell elongation-related genes *OsEXPB7*, *OsEXP13* and *OsXTH12* were significantly less repressed by JA treatment in *oscoi2-2* crown roots than in WT and in *oscoi1a-1/b-1* mutant background (Fig 2d). Conversely, expression of cell cycle or meristem marker genes (*OsCYCB1*, *OsCDKB1*, *OsCDKB2*, *OsPLT1* and *OsPLT6*) was not affected by JA treatment [25]. Furthermore, genes associated with defence responses (*OsPR5*, *OsPR10* and *OsWRKY71*) were significantly less induced by JA treatment in *oscoi2-2* than in WT and *oscoi1a-1/b-1*. Hence, expression profiling revealed that *OsCOI2*, and not *OsCOI1a/b*, is required for JA-dependent signalling in rice crown root.

## Discussion

Here, we report that a monocot-specific member of the COI family *OsCOI2* mediates the regulation of a range of jasmonate-dependent vegetative and reproductive developmental processes. Significantly, the loss of *OsCOI2* function (and not that of *OsCOI1a* and *OsCOI1b* genes) was shown to strongly alter male fertility in rice, a phenotype that is *AtCOI1*-dependent in Arabidopsis. In rice, jasmonate is not only necessary for stamen fertility, but also required for floret development [26, 27]. Loss-of-function mutations or misexpression in key genes in the jasmonate biosynthetic or signalling pathways cause floral morphological alterations such as longer sterile lemmas and extra glume-like organs [24, 26, 27]. We found that *oscoi2* anthers did not dehisce properly, a phenotype also observed in the rice *osjar1* mutant impaired in JA-Ile biosynthesis [28]. Furthermore, previous reports showed that *OsCOI2* does not restore fertility and JA signal transduction in the Arabidopsis *coi1* mutant, unless the *OsCOI2* His-391 was substituted with Tyr-391 [13]. Recently, two reports also described that *oscoi2* mutant anthers do not dehisce properly [29, 30]. In addition, Wang et al., 2023 showed that the pollen germination rate of *oscoi2* mutants was significantly lower than that of WT plants. Collectively, these data point to a specialized function of COI2 in the regulation of plant reproductive development in the Monocot phylum.

*COI1* was shown to be the receptor of the jasmonate signal in Arabidopsis and in rice. We investigated the possible role of *OsCOI2* in jasmonate signalling using the JA-mediated repression of crown root growth. Surprisingly, our data suggested that inhibition of crown root growth by JA relies primarily on *OsCOI2*. Although the roots of *oscoi2* mutant plants showed better resistant to JA than that of *oscoi1a* and *oscoi1b* double mutant, its growth is still inhibited to some extent suggesting the minor contribution of OsCOI1a and OsCOI1b. Our observation is also in line with the report in Inagaki et al., (2023) and Wang et al., (2023) [29, 30], although the sensitivity to JA treatment varies to some extent between studies probably due to the differences in genetic background, alleles chosen, type of roots, and/or treatments. Consistently, expression profiling revealed that *OsCOI2* is specifically required for JA-dependent signalling in crown roots. This suggests that sub-functionalisation of JA receptors has occurred in the monocot lineage.

Besides its role in crown root growth inhibition, jasmonate is a potential regulator of adventitious root initiation. For example, JA treatment inhibited adventitious root formation in Arabidopsis hypocotyl [31]. Accordingly, *atcoi1-1* mutant produced more adventitious root compared to the WT indicating a negative role of JA in this developmental process [31]. Here, our *oscoi2* mutants also produced adventitious root on the above-ground nodes, a specific phenotype that has not been reported by Inagaki et al., (2023) and Wang et al., (2023) [29, 30]. In rice, adventitious root emergence is inducible by flooding and is tightly regulated by various hormones such as ethylene and auxin [32, 33]. Constitutive formation of adventitious roots from *oscoi2* nodes suggests that this non-canonical jasmonate receptor could be a central regulator of shoot-borne root development in rice.

Specific functions of *OsCOI2* could derive from (i) its spatial expression pattern being different from *OsCOI1a/b*, and/or (ii) distinct protein-protein interactions involving a subset of OsJAZs, and/or (iii) the perception of distinct jasmonate forms. For instance, the liverwort *Marchantia polymorpha* MpCOI1 receptor was shown to interact with distinct hormone forms that the eudicot Arabidopsis COI1 receptor did not bind [34]. Specifically, instead of perceiving JA-Ile, MpCOI1 binds the JA precursor dn-OPDA as an ancestral ligand to initiate jasmonate signalling [34]. In rice, despite of JA-Ile functions being established, OPDA- and distinct jasmonic acid- amino acid conjugates have been shown to be involved in defence response and could be additional ligands of OsCOI proteins [35, 36]. OsCOI2 was shown to bind with different jasmonate derivatives and to interact with OsJAZ proteins with some selectivity compared to OsCOI1a and OsCOI1b JA receptors [29, 30]. This could explain the specificity of OsCOIs function.

In summary, we report that COI jasmonate receptor sub-functionalisation has occurred in rice, consistent with the hypothesis that different bioactive forms of jasmonate could modulate distinct responses to developmental cues and environmental stresses via *OsCOI1a/b* and *OsCOI2* perception. Our collection of *oscoi* mutant alleles offers a unique genetic resource for future dissection of the function of jasmonate receptor proteins in monocots.

## Supporting information

S1 File (PDF)
