# Supplementary material for: Genetic analysis of the rice jasmonate receptors reveals specialized functions for OsCOI2
Source: PLoS One. 2023 Sep 8;18(9):e0291385. doi: 10.1371/journal.pone.0291385 (PMC10490909; doi:10.1371/journal.pone.0291385)
Supplement: S1 File — (PDF) [file pone.0291385.s001.pdf]

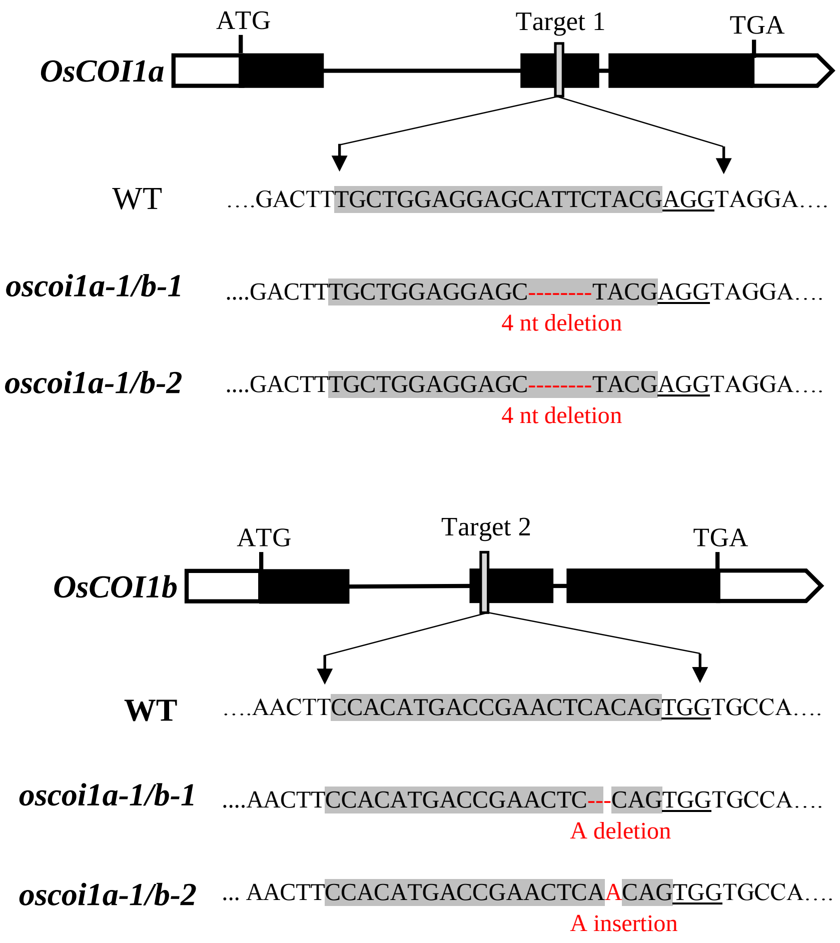

Figure S1. Schematic representation of the *oscoi1ab* double mutant lines.
OsCOI1a and OsCOI1b gene structures and the CRISPR-Cas9 target sites are shown. The insertion and deletion sites of two allelic mutations (*oscoi1a-1/b-1*, *oscoi1a-1/b-2*) generated by two gRNAs are shown in comparison with the wild-type (WT) sequence.

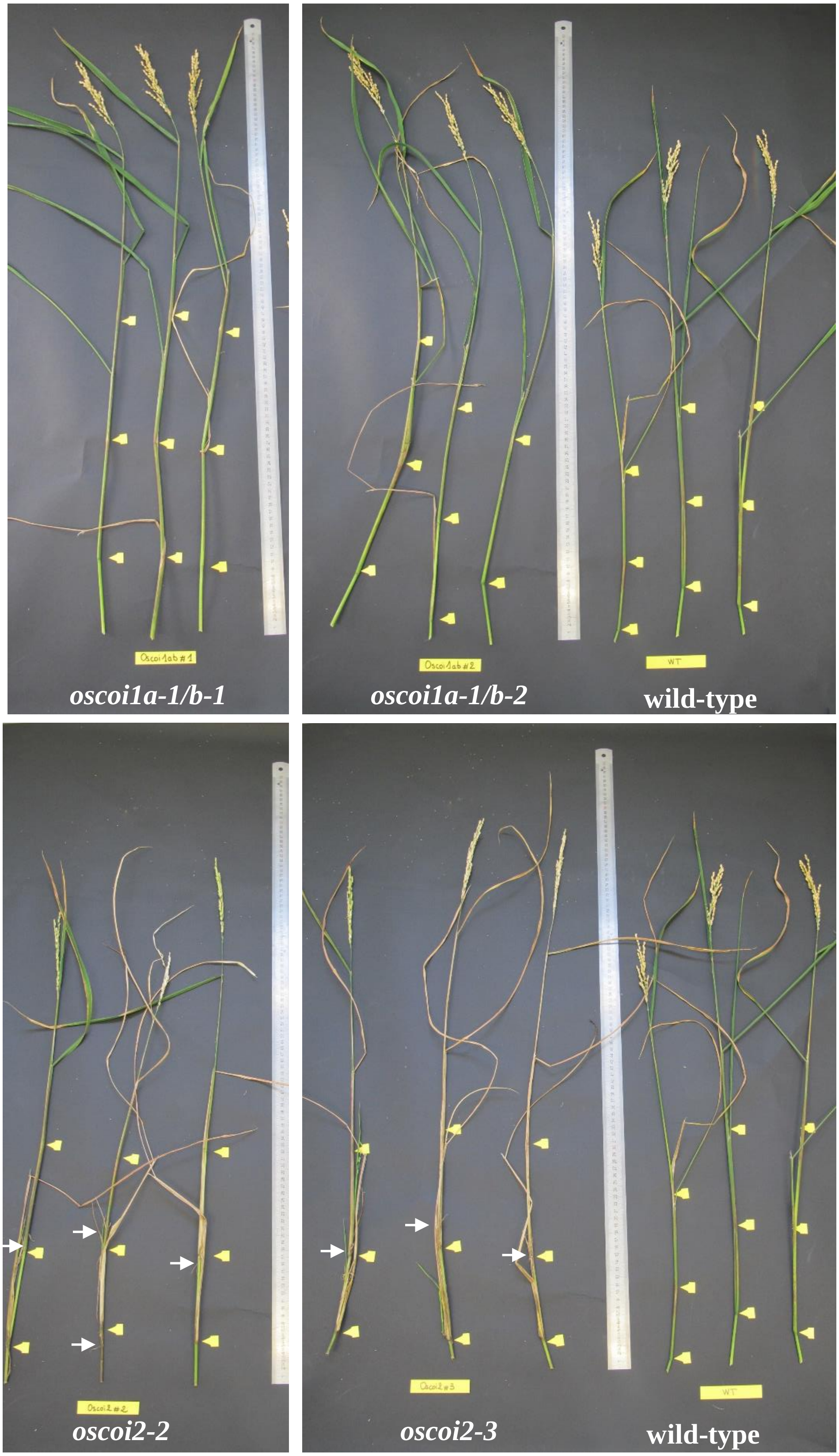

Figure S2. Images of wild type, *oscoi1a-1/b-1*, *oscoi1a-1/b-2*, *oscoi2-2* and *oscoi2-3* show panicule phenotypes.

Yellow arrows show nodes 1, 2 and 3. White arrows indicate adventitious roots from nodes.

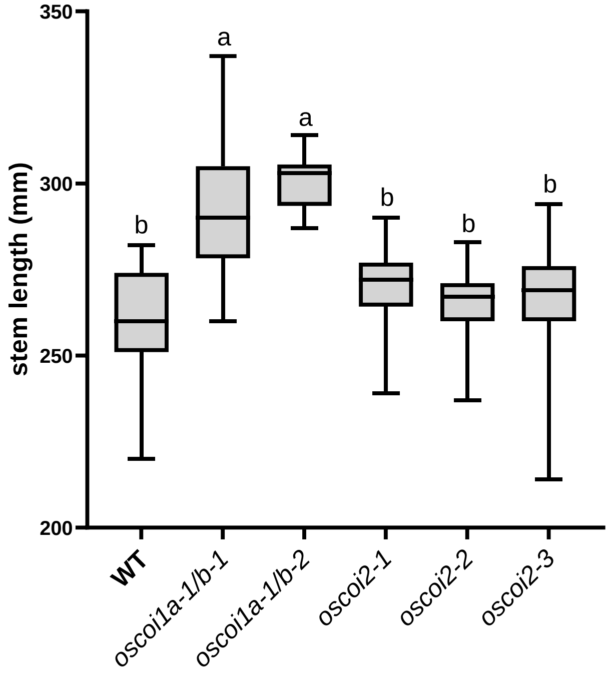

Figure S3. Quantification of stem length of the WT and *oscoi* mutant plantlets.

Quantification of stem length of the WT and *oscoi* mutants plantlets. Letters indicate significant differences between lines (21<n<24, One way-ANOVA with Tuckey's multiple comparisons test, p<0.05). In the boxplots, whiskers denote minimum/maximum values, the box defines the interquartile range and the centre line represents the median.

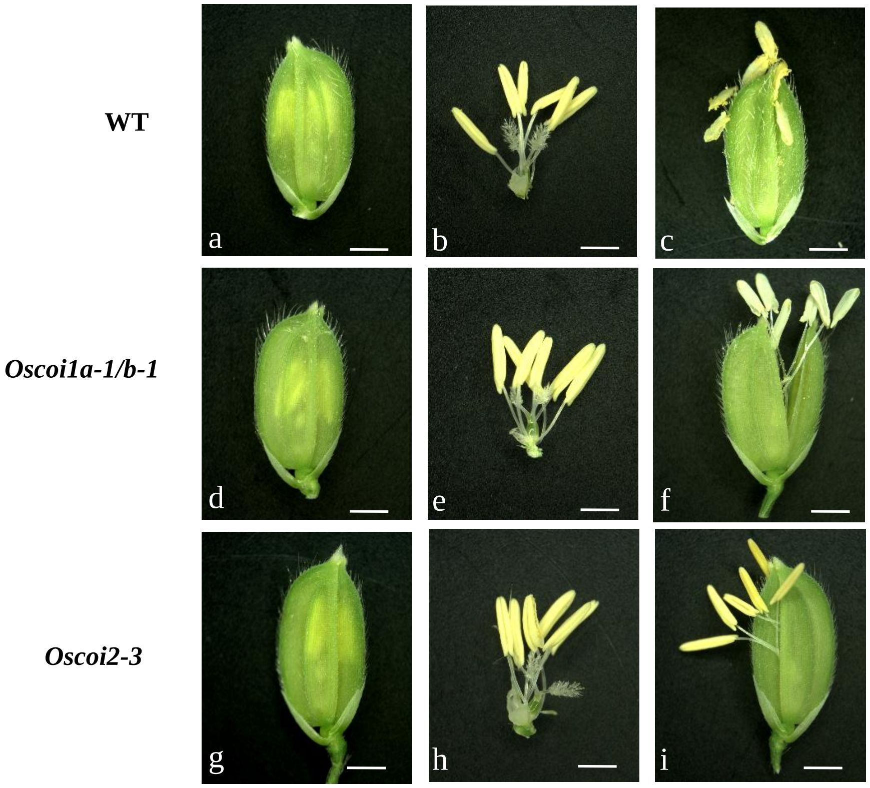

Figure S4. Comparison of anther dehiscence between the wild-type and the *oscoi* mutants.
Close spikelet before anthesis (a, b ,d, e, g and h) and after widely opened spikelet (c, f, i).

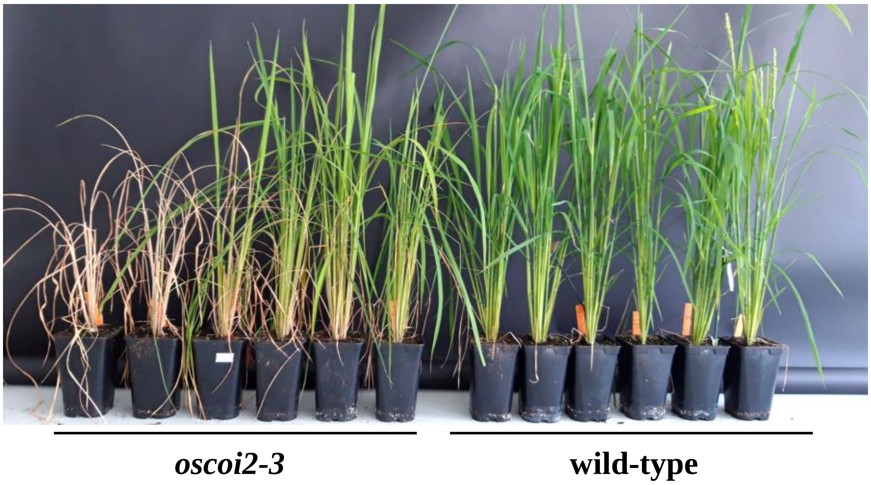

Figure S5. Image of wild-type and *oscoi2-3* mutant plants showing mortality at reproductive stage.

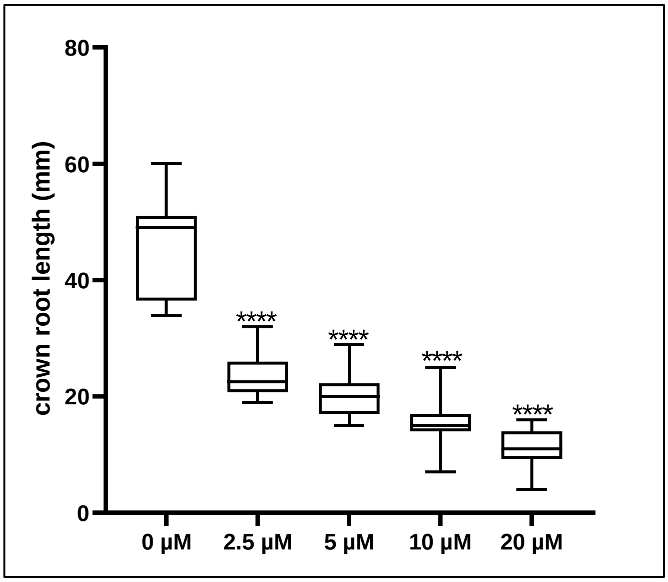

Figure S6. JA dose-dependent effect on crown root length

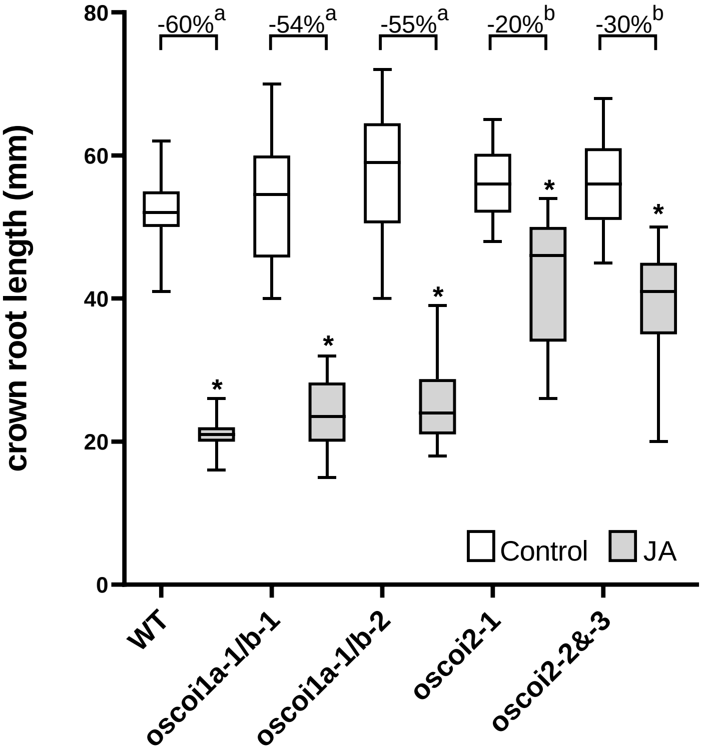

Figure S7. Crown root growth inhibition assay by JA application on WT and T2 *oscoi* mutants. Effect of JA on crown root length of the WT, two allelic mutations of the double mutant *oscoi1a*/*oscoi1b* (*oscoi1a-1/b-1* and *oscoi1a-1/b-2*) and two mutants of *OsCOI2* (*oscoi2-1*, *oscoi2-2&*-3 heterozygous line). In the boxplots, whiskers denote minimum/maximum values, the box defines the interquartile range and the center line represents the median. Asterisks indicate significant differences between treated and control plants (One way-ANOVA with Bonferroni's multiple comparisons test, $p<0.05$).The plot values are the means of the crown roots. Percentage of crown root growth inhibition by JA from the WT, *oscoi1a-1/b-1*, *oscoi1a-1/b-2*, *oscoi2-1* and *oscoi2-2&*-3 heterozygous line. Letters (a, b) indicate significant differences between lines (One way-ANOVA with Tuckey's multiple comparisons test, $p<0.05$)

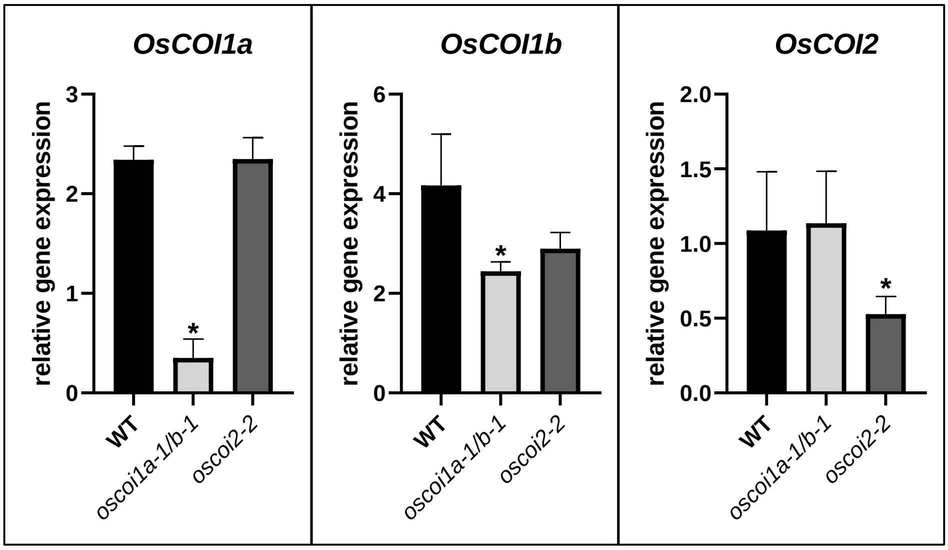

Figure S8. Relative expression of *OsCOI* genes in WT and *oscoi* mutants in crown root tips

Gene name	Locus or Target	Forward primer	Reverse primer	Amplicon size	Use
OsCOI1a	LOC_Os01g63420	GATGCCCTCCCTGAGATACA	CCACACAGGGTTCTCCATCT	158 bp	qPCR
OsCOI1b	LOC_Os05g37690	CAGGCCTTGGCTATATTGGA	CAGGGAAGGCATACTCCGTA	188 bp	qPCR
OsCOI2	LOC_Os03g15880	ATGGGTGCAAGGATACAAGG	TGCAAGAATCTGTGCCTGAC	133 bp	qPCR
OsAOC	LOC_Os03g32314	AAGAGGAATCGAGGACAAGATATTTG	AAGCCTCTTCTTGTTCGGATCA	75 bp	qPCR
OsJAZ5	LOC_Os04g32480	CGAGGCAACTAAAGCAAAAGGA	TGAGTGGCTCTTTGGCAAAATT	71 bp	qPCR
OsJAZ8	LOC_Os09g26780	GAAGGCTCAACAGCTGACCAT	TTGGTGGACGGGAAGTTCTC	69 bp	qPCR
OsMYC2	LOC_Os10g42430	CTAGCGAGGAAACCCAATCG	CCATCCATCCATCCTAACAC	158 bp	qPCR
OsWRKY71	LOC_Os02g08440	CGGCAAAAGACGCTTGTAAC	TCGGGACCGAAGCAAATTTG	106 bp	qPCR
OsPR5	LOC_Os12g43380	CTGGCGGAGTTCACCATC	GCAGGAGAAGCTCATGGC	87 bp	qPCR
OsPR10	LOC_Os03g18850	CGGACGCTTACAACTAAATCG	AAACAAAACCATTCTCCGACAG	174 bp	qPCR
OsPLT1	LOC_Os04g55970	AACATTCGGCACTGAGGAGG	CTCCCGATTGGAAGGTTGCT	141 pb	qPCR
OsPLT6	LOC_Os11g19060	TCCAGACGTCACAGGTTTCG	CGGGAGACCATGAAAGCCAT	94 pb	qPCR
OsEXPB7	LOC_Os03g01270	GTCAGTATCCAGGGCTGACG	CGTACTCCACCAGTATCGCC	81 bp	qPCR
OsEXP13	LOC_Os02g16730	CAAGTGAGCCACCTGATCGAT	ACCCCCATCAGCATCCAAATT	153 pb	qPCR
OsXTH12	LOC_Os06g48180	AGTGCACTGCCAAGTAGGAAA	TCTTCCTCGTATCCTTTTCCCA	133 pb	qPCR
OsCYCB1	LOC_Os01g59120	CTCTCAAGCACCACACTGGA	CTTGCTCTCAGGGGCAGTAG	92 pb	qPCR
OsCDKB1	LOC_Os01g67160	GTCCGTTGGTTGCATCTTCG	GTTACTCCAGGCCACTGCTC	132 pb	qPCR
OsCDKB2	LOC_Os08g40170	CGCTCGTTCACTGTCCCTCT	CCACAGACCAGATGTCAACCG	121 pb	qPCR
EXP	LOC_Os06g11070	TCCATCTGCTCCCGTTGTTGTG	AAAGAGTTCGCCACCAACCGTC	273 bp	qPCR
OsCOI1a	target 1	TGGAAGAGTGCCATATTACTG	TCAAACACAATTAATCAATGC	496 bp	identify mutations
OsCOI1b	target 2	TGTCATACTTTAGCTCACTGG	ACGTAAGTCCTAAGGAGCACA	477 bp	identify mutations
OsCOI2	targets 1 and 2	CCATGGCGTACTCCACCACG	GAGAGAACTCCTAAGACGTAG	462 bp	identify mutations
OsCOI2	targets 3 and 4	CCATATGTTGTCATTTGCAG	CAGCGCTGGACCAGCTGACAG	485 bp	identify mutations
Cas9	T-DNA	GGATGATGGCATATGCAGCAG	GAGTGTGAGGTCCTGGTGGT	1216 bp	check T-DNA insertion
HPT	T-DNA	CTCGGAGGGCGAAGAATCTC	GCTCCAGTCAATGACCGCTG	759 bp	check T-DNA insertion

Table S1. List of primers used for qPCR and plant genotyping.
